# Pregnancy Epulis

**DOI:** 10.31662/jmaj.2022-0156

**Published:** 2023-02-27

**Authors:** Yoshitsugu Chigusa, Ryuji Okuda, Yukina Teratani, Sunao Matsuzaka, Masaki Mandai

**Affiliations:** 1 Department of Gynecology and Obstetrics, Graduate School of Medicine, Kyoto University, Kyoto, Japan; 2 Department of Oral and Maxillofacial Surgery, Graduate School of Medicine, Kyoto University, Kyoto, Japan

**Keywords:** epulis, granuloma, pregnancy

A 29-year-old primigravida was presented with intraoral bleeding at the 38^+0/7^ weeks of gestation. A granuloma-like mass of about 23 mm with bleeding was found in left maxillary gingiva (Figure 1). This lesion had arisen during her pregnancy and had grown rapidly and repeatedly bled in the past month. The bleeding stopped after 20 min of pressure by the dentist. The same day, she underwent emergent cesarean section due to nonreassuring fetal status. Thirty-six days postpartum, the tumor was resected (Figure 2). Microscopically, the lesion was lined by stratified squamous epithelium and comprised prominent hypervascularization and fibrous connective tissues with inflammatory cell infiltration, indicating pregnancy epulis (Figure 3). The postoperative course was uneventful (Figure 4). Pregnancy epulis is a hyperplastic lesion originating from oral mucosa ^(1), (2)^. The excision is not recommended during pregnancy because of the risk of heavy bleeding and the possibility of regression after delivery. However surgical resection should be considered if it does not shrink even after childbirth. Complete excision and subsequent maintenance of oral hygiene are important to prevent recurrence ^(3)^.

**Figure 1.**
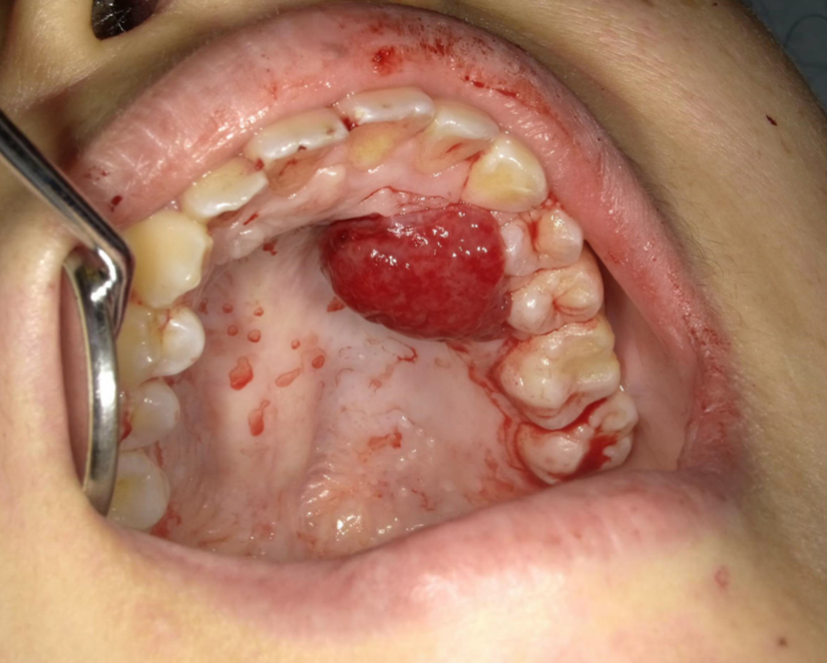
Intraoral image at the time of admission. A granuloma-like mass with irregular surface and bleeding was found.

**Figure 2.**
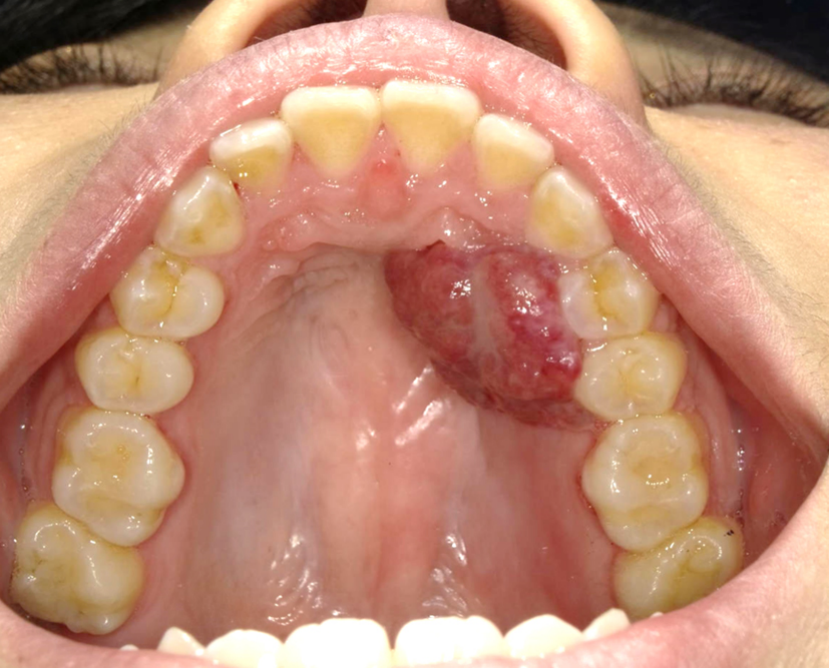
Intraoral image at the time of surgery. The lesion did not shrink spontaneously after childbirth.

**Figure 3.**
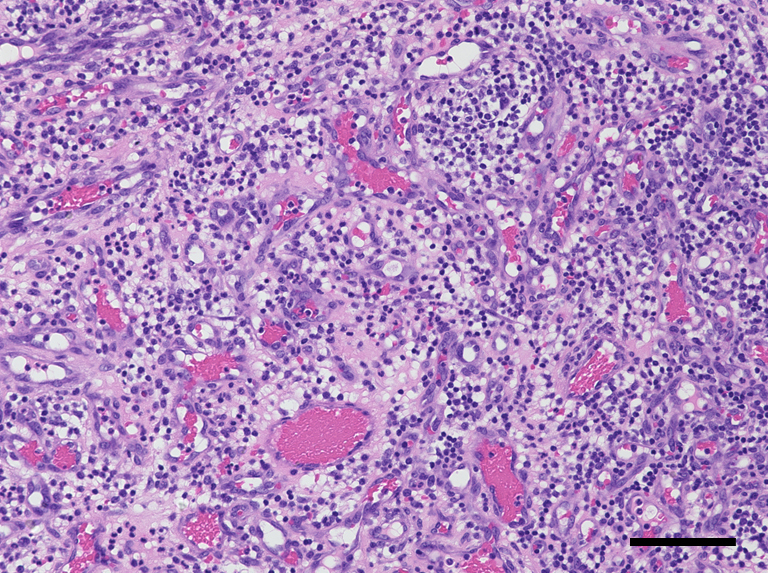
Microscopic findings of the specimen. The lesion was lined by stratified squamous epithelium, and consisted of prominent hypervascularization and fibrous connective tissues with inflammatory cell infiltration. Hematoxylin and eosin staining, bar 100 μm, ×200.

**Figure 4.**
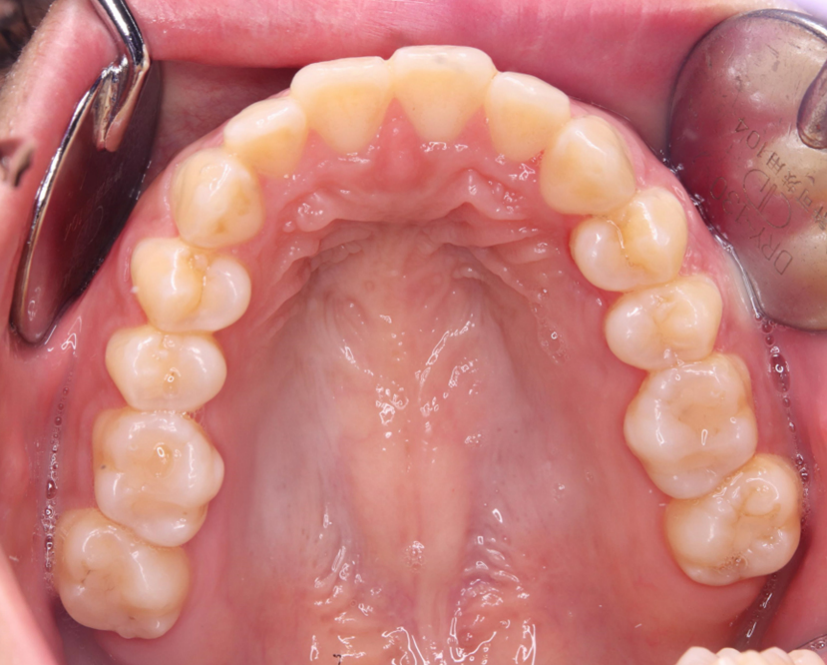
Intraoral image at one month postoperatively. Wound healing was satisfactory.

## Article Information

### Conflicts of Interest

None

### Acknowledgement

We would like to thank Editage (www.editage.com) for English language editing.

### Author Contributions

RO, YT, and SM contributed to patient care, and collected patient’s data and images. YC wrote the manuscript, and the other authors revised it. MM supervised the entire process.

### Approval by Institutional Review Board (IRB)

This study did not require IRB approval.

### Informed Consent

The written informed consent was obtained from the patient to publish this case, including pictures.
